# Supplementary figures and images for: Tradeoff between metabolic i-proteasome addiction and immune evasion in triple-negative breast cancer
Source: Life Sci Alliance. 2020 May 18;3(7):e201900562. doi: 10.26508/lsa.201900562 (PMC7240743; doi:10.26508/lsa.201900562)

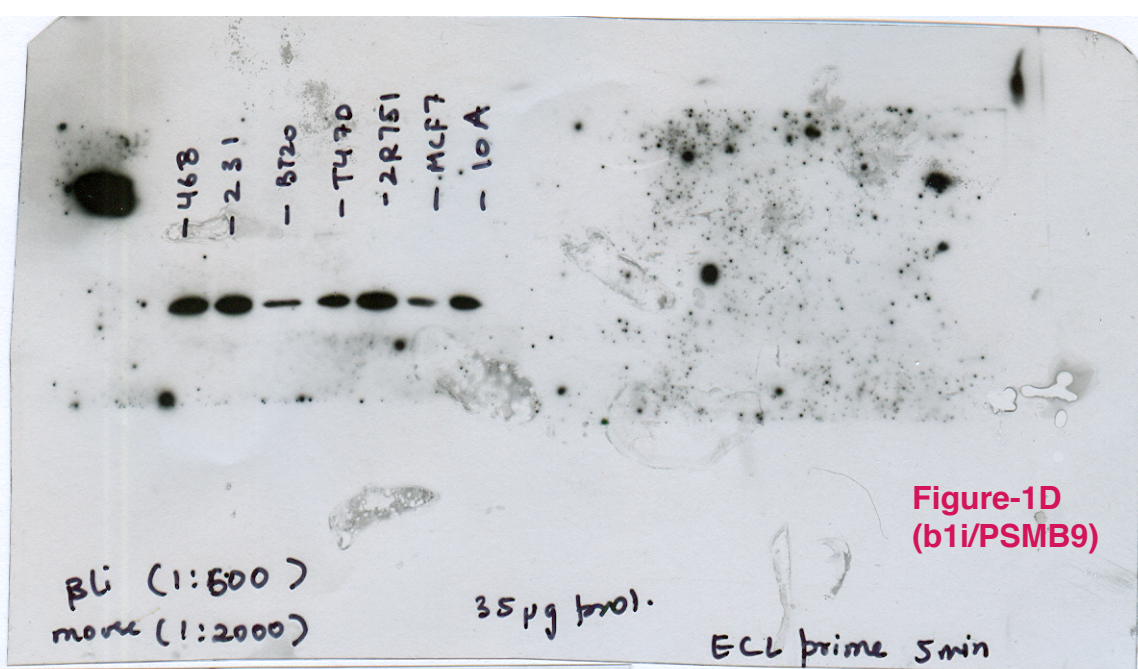

Figure-1D  
(b1i/PSMB9)

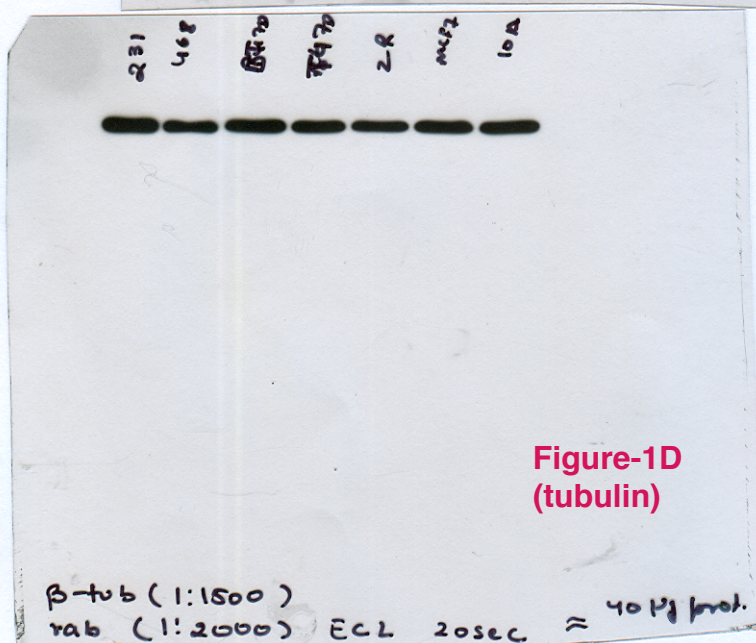

Figure-1D  
(tubulin)

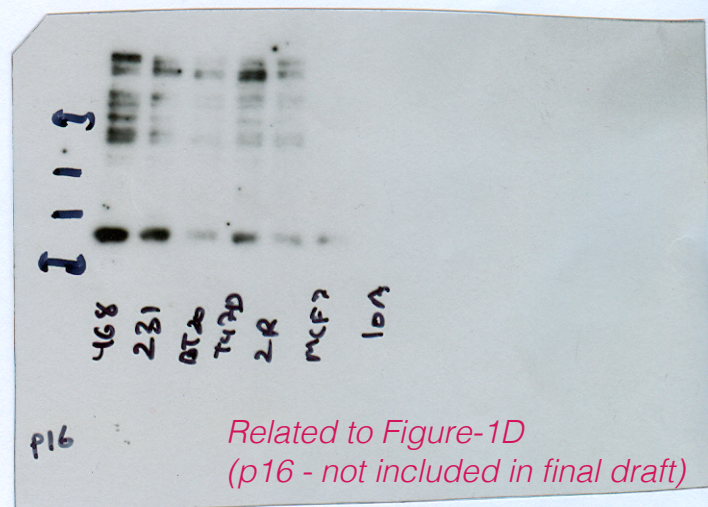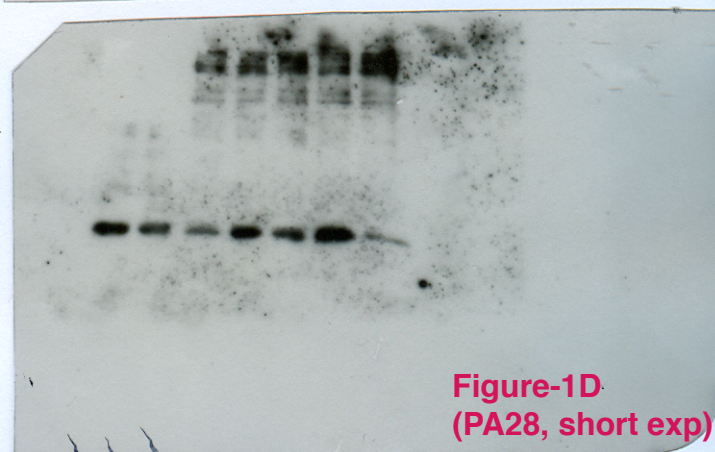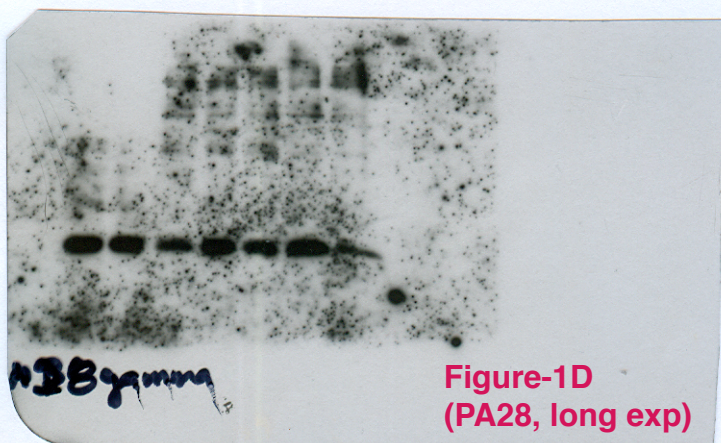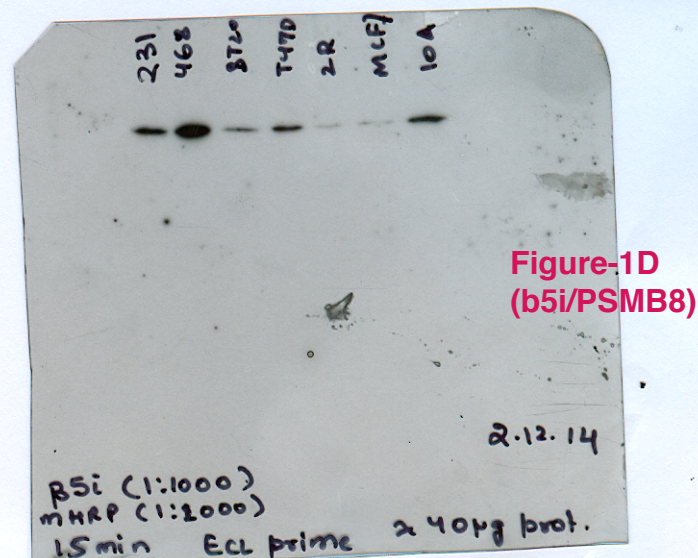

Figure-1D  
(b5i/PSMB8)

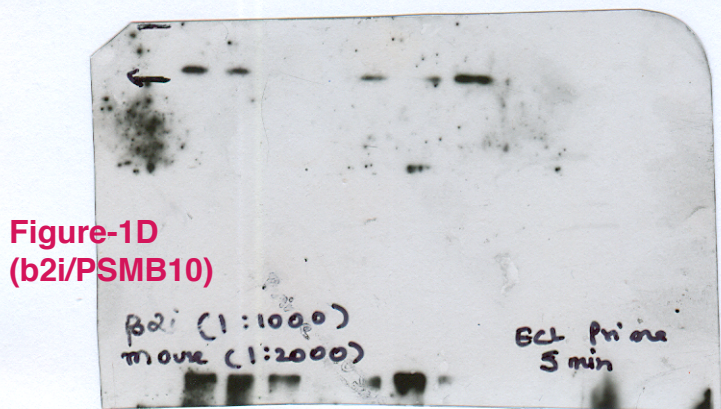

Figure-1D  
(b2i/PSMB10)

Supplement: Supplementary file 1 [file LSA-2019-00562_SdataF1.pdf]
